# Quantitative Phosphoproteomics Reveals System-Wide Phosphorylation Network Altered by Spry in Mouse Mammary Stromal Fibroblasts

**DOI:** 10.3390/ijms20215400

**Published:** 2019-10-30

**Authors:** Tiezhu Shi, Linli Yao, Ying Han, Piliang Hao, Pengfei Lu

**Affiliations:** 1 School of Life Science and Technology, ShanghaiTech University, Shanghai 201210, China; shitzh@shanghaitech.edu.cn (T.S.); yll0123456@126.com (L.Y.); hanying@shanghaitech.edu.cn (Y.H.); haopl@shanghaitech.edu.cn (P.H.); 2 CAS Center for Excellence in Molecular Cell Science, Shanghai Institute of Biochemistry and Cell Biology, Chinese Academy of Sciences, Shanghai 200031, China; 3 University of Chinese Academy of Sciences, Beijing 100049, China

**Keywords:** sprouty, receptor tyrosine kinase signaling pathway, quantitative phosphoproteomics, mammary stromal microenvironment

## Abstract

Understanding the fundamental role of the stroma in normal development and cancer progression has been an emerging focus in recent years. The receptor tyrosine kinase (RTK) signaling pathway has been reported playing critical roles in regulating the normal and cancer microenvironment, but the underlying mechanism is still not very clear. By applying the quantitative phosphoproteomic analysis of Sprouty proteins (SPRYs), generic modulators of RTK signaling and deleted mouse mammary fibroblasts, we quantified a total of 11,215 unique phosphorylation sites. By contrast, 554 phosphorylation sites on 425 proteins had SPRY-responsive perturbations. Of these, 554 phosphosites, 362 sites on 277 proteins, were significantly increased, whereas 192 sites on 167 proteins were decreased. Among the regulated proteins, we identified 31 kinases, 7 phosphatases, and one phosphatase inhibitor that were not systematically characterized before. Furthermore, we reconstructed a phosphorylation network centered on RTK signaling regulated by SPRY. Collectively, this study uncovered a system-wide phosphorylation network regulated by SPRY, providing an additional insight into the complicated RTK signaling pathways involved in the mammary gland microenvironment.

## 1. Introduction

The local microenvironment is essential in regulating cell polarity, migration, cell differentiation and tumor progression [1,2,3]. Fibroblasts constitute a major component of the stroma, and are the predominant cells in the crosstalk between epithelial cells and their local niche [4]. It has been shown that fibroblasts affect cell behaviors through paracrine effects, and therefore establish a specific extracellular matrix (ECM) for tissue formation and tumorigenesis [5,6,7]. In the mammary gland, stromal-epithelial interactions play a fundamental role in determining normal duct initiation and elongation and the maintenance of hormone responsiveness in mammary epithelial cells [8]. Mammary gland fibroblasts secrete ECM constituents, such as fibronectin, tenascin and matrix metalloproteinases (MMPs), which affects cell adhesion, cell proliferation, inflammation and ECM degradation [9,10].

Indeed, genetic and epigenetic changes in stromal fibroblasts are one of the important hallmarks of cancer, which is becoming considerable in cancer prognosis and therapy [9,11,12], including breast cancer [13,14].

The receptor tyrosine kinase signaling pathway has been widely studied in developmental and cancer biology for decades [15,16]. In recent years, several small proteins that regulate this RTK signaling activity have been identified, including the Sprouty (Spry) family members: SPRY1, 2, 3 and 4. Spry was first uncovered as an inhibitor of the *breathless*, equivalent of the fibroblast growth factor (FGF) receptor, in *Drosophila melanogaster*.

Previous studies have reported that SPRY proteins function as both negative and positive regulators of Ras, extracellular signal–regulated kinase (ERK) and mitogen-activated protein kinase (MAPK) signaling pathways that are downstream of several RTK signaling (FGF, epidermal growth factor (EGF), platelet-derived growth factor (PDGF) and vascular endothelial growth factor (VEGF)) pathways in a cell type-dependent manner (Table 1). It has been recognized that RTK signaling plays an important role in regulating the activity of epithelial cells and cancer cells; however, the function of RTK signaling in regulating stromal cells, particularly fibroblasts, remains unclear. Our previous study revealed that SPRY1 inhibits the epidermal growth factor receptor (EGFR)-dependent stromal paracrine signaling pathway and ECM remodeling, by decreasing ERK signaling activity in mammary stromal fibroblasts. Notably, in the study, overexpression of *Spry2* in mammary stroma has contrary phenotypes compared with *Spry1* loss [6]. This data indicates that similar to the SPRY function in other cellular contexts [16,17,18,19], SPRY family proteins may play redundant roles in the mammary stromal fibroblasts. 

In cancer evolution, it has been reported that stromal cells expressing RTK downstream molecules can be recruited to the metastatic site to form a pre-metastatic niche through RTK-regulated signaling [20,21]. However, the roles that RTK signaling performs in stromal cells, especially in fibroblasts, is still not very clear. SPRY is the recognized generic modulator of RTK signaling. To unveil the signal transduction, perturbed by SPRY loss, downstream of RTK signaling in mammary stromal fibroblasts, we applied Tandem Mass Tag (TMT)-labeled and large-scale liquid chromatography-tandem mass spectrometry (LC-MS/MS)-based phosphoproteomic profiling and quantification in mouse mammary stromal fibroblasts.

## 2. Results

### 2.1. Phosphoproteomic Profiling and Quantification of Sprouty (SPRY)-Deficient Mouse Mammary Stromal Fibroblasts

Sprouty (Spry) family genes Spry1, Spry2 and Spry4 are differentially expressed in breast cancer, but Spry3 is rarely expressed [22,23]. This result indicates that Spry1, 2 and 4 are the predominant orthologs in the mammary gland. In our previous study, we showed that Spry1 regulates normal mammary branching morphogenesis through modulating EGFR-dependent paracrine signaling and remodeling ECM in stromal fibroblasts. We also reported that the phenotypes of Spry2 overexpression in the mammary stroma are in contrast to Spry1 loss [6]. These data implicated that Spry family proteins play redundant roles in mammary gland stroma.

**Figure 1:**
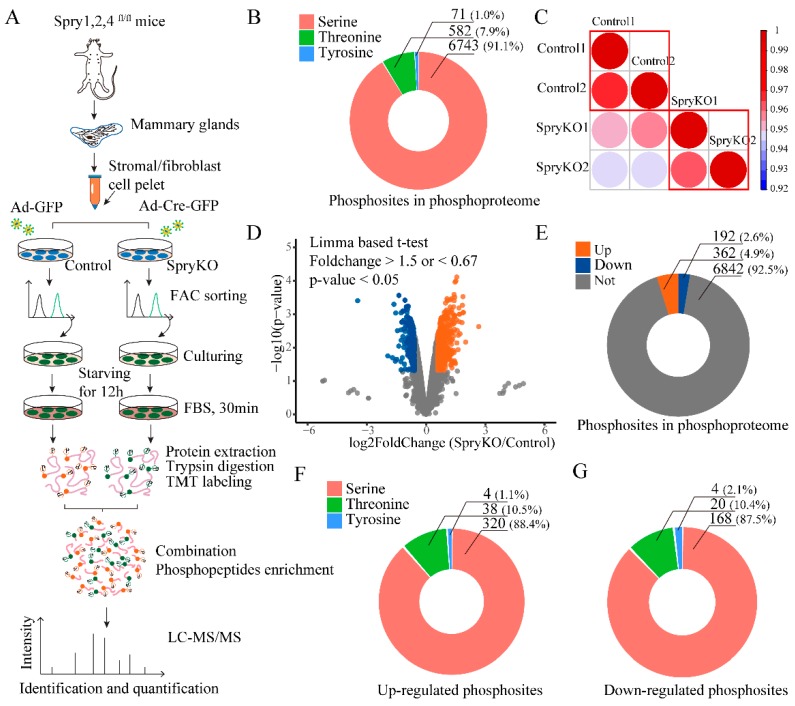
Overview of the analysis of SPRY-induced phosphoproteome in *SpryKO* mouse mammary stromal fibroblasts. (**A**) Schematic of phosphoproteome analysis workflow for the study of phosphorylation induced by a SPRY deficiency in mammary stromal fibroblasts. Two biological replicates were performed in the control and *SpryKO* groups, respectively; (**B**) Distribution of serine, threonine and tyrosine phosphorylation residuals in total quantified phosphosites with high localization probability (>0.75). Numbers and percentages are as shown in the ring plot; (**C**) The Pearson correlation coefficient between the duplicate mass spectrometry results in control and *SpryKO* mammary stromal fibroblasts; (**D**) Volcano plot of differently-regulated phosphosites by the SPRY deletion in mammary stromal fibroblasts. Limma-based *t*-test was used to determine the statistical significance in the analysis. Downregulated sites: Blue points, *p*-value < 0.05, fold change < 0.67; up-regulated sites: Orange points, *p*-value < 0.05, fold change > 1.5; (**E**) Phosphorylation residuals distribution of the differently regulated phosphosites. Numbers and percentages are as shown in the ring plot; (**F**) Phosphorylation residuals distribution of the up-regulated phosphosites. Numbers and percentages are as shown in the ring plot; (**G**) Phosphorylation residuals distribution of the down-regulated phosphosites. Numbers and percentages are shown in the ring plot.

To further elucidate SPRY-dependent signaling events, we performed TMT-labeled quantitative phosphoproteome analysis of Spry1, 2 and 4 triple-knockout (*SpryKO*) mouse mammary stromal fibroblasts as illustrated in Figure 1A. Fetal bovine serum (FBS) is a widely used stimulant in investigating phosphorylation in ERK signaling and AKT signaling [24,25]. We collected phosphorylated proteins by starving fibroblasts for 12 h in culture medium without FBS, and then stimulating for 30 mins with 10% FBS. The overall analysis of FBS-stimulated *SpryKO* and control fibroblasts identified 11,215 unique phosphorylation sites on 3659 proteins in total. Of these 11,215 phosphorylation sites, 7396 sites were with high localization probability (>0.75) and 6762 (~90%) sites were known phosphosites in previous reports (Table S1). Among the 7396 phosphorylation sites, there are 6743 serine, 582 threonine, and 71 tyrosine. The proportions of phosphorylated serine, threonine and tyrosine residues in the total 7446 phosphosites are 91.1%, 7.9% and 1.0% (Figure 1B), respectively, which is quite consistent with previous reports [26,27]. Importantly, experiments based on synthetic libraries with similar abundances of the three phosphorylated amino acids, have revealed that the TiO_2_-based enrichment methods do not favor one type of the three phosphorylated residues [28]. The biological replicates demonstrated good reproducibility and uniformity, as evident from their correlation and distribution (Figure 1C and Figure S2A).

The limma-based *t*-test, a widely-used approach for the analysis of quantitative mass spectrometry data [29,30,31], was employed to determine statistical significance in this study. Using this approach, we identified that 554 phosphorylation sites on 425 proteins had statistically significant SPRY1/2/4-responsive alterations. Of these 554 phosphosites, 362 sites on 277 proteins were significantly increased (*p*-value < 0.05, fold change >1.5), whereas 192 sites on 167 proteins were decreased (*p*-value < 0.05, fold change < 0.67) (Figure 1D,E).

However, changes in phosphorylation signals are closely related to the expression level of phosphorylated proteins. To address whether the changes in phosphorylation by SPRY1, 2 and 4 triple knockout are due to changes in protein expression, we performed the TMT-labeled quantitative proteome analysis between control and SpryKO fibroblasts by LC-MS/MS, using the same experimental materials with the same treatment as the phosphoproteomics experiment. The differential expression analysis approaches of the total proteins are also the same. In the phosphoproteome analysis, we revealed 554 phosphorylation sites on 425 proteins that had SPRY-responsive perturbations (Figure 1D,E). However, of the 425 regulated proteins, only 337 proteins (79.3%) were identified in the total protein proteomic analysis, which is consistent with previous study. Importantly, by setting the same cutoff (*p*-value < 0.05 and fold change > 1.5 of < 0.67) as the phosphorylation sites analysis, none of the expression levels of the 337 proteins has significant change (Figure S2B). This result indicates that, in this study, the changes in phosphorylation of the proteins are regulated by SPRY genomic deletion rather than changes in protein expression. 

For the up-regulated and down-regulated phosphosites, the distributions of the three phosphorylated residues (serine, threonine and tyrosine) are as shown in Figure 1F,G. Four tyrosine sites were up-regulated, and four other sites were down-regulated, respectively. The up-regulated phosphorylated tyrosine sites include MAP1B (Y1792) [32], MAPK3/ERK1 (Y205) [6,33], SHC1 (Y423) [34] and STAT5B (Y699) [35], and they are all known phosphorylation sites reported previously. However, of the four down-regulated phosphorylated tyrosine sites i.e., ARHGEF40 (Y759), KLHL32 (Y434), MYOT (Y461) and ZBTB11 (Y319), only one site ARHGAP42 (Y759) [36] was reported (Table S2).

### 2.2. The Functional Enrichment Analysis of SPRY-Altered Phosphoproteins in Mammary Stromal Fibroblasts

**Figure 2:**
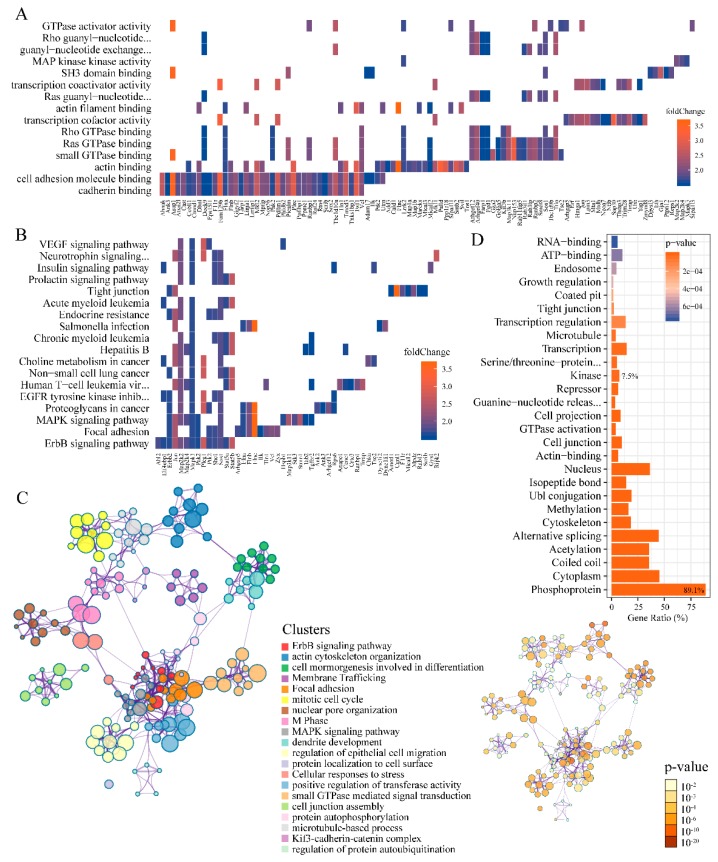
The functional enrichment of SPRY-altered phosphoproteins in mouse mammary stromal fibroblasts. (**A**) Heat plot of Gene Ontology (GO)-enriched molecular function of the up-regulated phosphoproteins. The color of the blocks indicates the fold change of each enriched protein; (**B**) Heat plot of Kyoto Encyclopedia of Genes and Genomes (KEGG) pathway enrichment of the up-regulated phosphoproteins. The color of the rectangle indicates the fold change of each enriched protein; (**C**) The complex network of enriched terms by Metascape, where terms with a similarity more than 0.3 are connected by edges. Nodes that share the same cluster ID are in the same color, and typically close to each other. The *p*-value of each term is also shown; (**D**) Protein class distribution of phosphorylation altered proteins. The phosphoproteins (370) occupy 89.1% and kinases (31) occupy 7.5%. The single-item enrichment *p*-value less than 0.001 are shown and ranked by *p*-value.

While the function of SPRY in mediating RTK signal transduction is widely reported (Table 1), a system-wide molecular network regulated by SPRY has only been realized in the past few years [37,38]. To further elucidate this, we conducted ontology-based enrichment of the modulated phosphoproteins in the phosphoproteome. Gene Ontology (GO) enrichment analysis of the molecular function of up-regulated phosphoproteins showed that the major cluster is cell adhesion molecule binding proteins (Figure 2A and Figure S2C).

We also observed site-specific upregulation on proteins relevant to the Ras GTPase superfamily activities, including Ras, Rho, and Rab GTPase binding proteins and GTPase regulator/activator activity proteins (Figure 2A and Figure S2C). Indeed, previous studies have reported that SPRY serves as a negative regulator of GTPase activity and Ras protein signal transduction [39,40], but the underlying mechanism remains undetermined. In the SPRY-deficiency-dependent phosphoproteome, we showed that several RAS oncogene family interacting proteins with known phosphorylated sites in RAB11FIP5 (S640), RAB3IL1 (S180) and RAB3IP (S218) were enriched in Ras GTPase binding function (Figure 2A, Table S2). These proteins were previously reported in regulating the process of GTPases transport and localization to intracellular membranes [41]. In addition, a significant increase in phosphorylation was categorized in SOS1 (S1123, S1122 and S1120), SMCR8 (S416), ARHGEF40 (T1515), FARP1 (S23), ARHGEF12 (S190), FGD1 (S135), DOCK9 (S20), TBC1D10A (S73 and S74) and TRIO (S2462) (Figure 2A, Table S2). These proteins were reported to be involved in modulating the activity of guanyl-nucleotide exchange factors (GEFs) that activate monomeric GTPases by stimulating the release of guanosine diphosphate (GDP) to allow the binding of guanosine triphosphate (GTP) [42]. Our results shed light on SPRY’s possible novel associations in influencing GTPase activity and Ras protein signal transduction by toggling GTPase from the inactive state (GDP-bound form) to the active state (GTP-bound form) to transduce signals.

Next, we looked into the signaling pathway classification of the up-regulated phosphorus proteins based on the Kyoto Encyclopedia of Genes and Genomes (KEGG) pathway. As expected, several RTK signaling pathways, including the Erb-B2 Receptor Tyrosine Kinase (ErbB) signaling pathway, insulin signaling pathway and VEGF signaling pathway, were enriched, which is in line with previous understanding of the major roles of SPRY in regulating RTK signaling pathways. Besides, the MAPK signaling pathway and the phosphatidylinositol 3’ -kinase (PI3K)-Akt (PI3K-Akt) signaling pathway, the two most important branches of the RTK signaling cascades, were also enhanced upon SPRY deletion (Figure 2B and Figure S3A). Increased phosphorylation of eight proteins related to EGFR tyrosine kinase inhibitor resistance were identified: PLCG1 (S1221), SOS1(S1123, S1122 and S1120), EIF4EBP1 (T69), MAPK3 (Y205), MAP2K2 (S226), RPS6 (S236), ERBB2 (S1103 and S1108) and SHC1 (Y423). The increased phosphorylation of Y205 on MAPK3/ERK1 is consistent with our previous study of SPRY1 in mouse mammary stromal fibroblasts [6]. Interestingly, our data also unveiled relationships between SPRY and the mammalian Target of Rapamycin (mTOR) signaling pathway with several members, including AKT1S1/PRAS40. Most of the known functions of AKT1S1/PRAS40 are dependent on its phosphorylation [43]. In our dataset, we revealed an increased phosphorus residue S214 on AKT1S1/PRAS40 which is not previously reported. It has been reported that AKT1S1/PRAS40, a subunit of mTOR1, can inhibit mTOR activity and suppresses the constitutive activation of mTOR in controlling cell growth and metabolism in responding to nutrients and growth factors [44]. Since AKT1S1/PRAS40 is an mTOR binding partner which can mediate AKT signals to mTOR, our results implicate that this process may be partially SPRY-dependent.

To further elucidate the relationships between the enriched terms of regulated phosphoproteins, a more comprehensive enrichment analysis was performed with the following defined ontology sources: GO Biological Processes, KEGG Pathway, Reactome Gene Sets and the comprehensive resource of mammalian protein complexes (CORUM) (Figure 2C, Table S3). The *p*-value of each enriched term is also shown in Figure 2C. In addition, GO biological process and cellular components analysis of down-regulated phosphoproteins showed that junction proteins and proteins associated with microtubule polymerization, actin filament-based movement and cell migration, are enriched (Figure S3B). These are consistent with the known function of Spry in the positive regulation of cell migration [45].

### 2.3. Kinases, Phosphatases, and Phosphatase Inhibitors Regulated by SPRY in Mammary Stromal Fibroblasts

**Figure 3:**
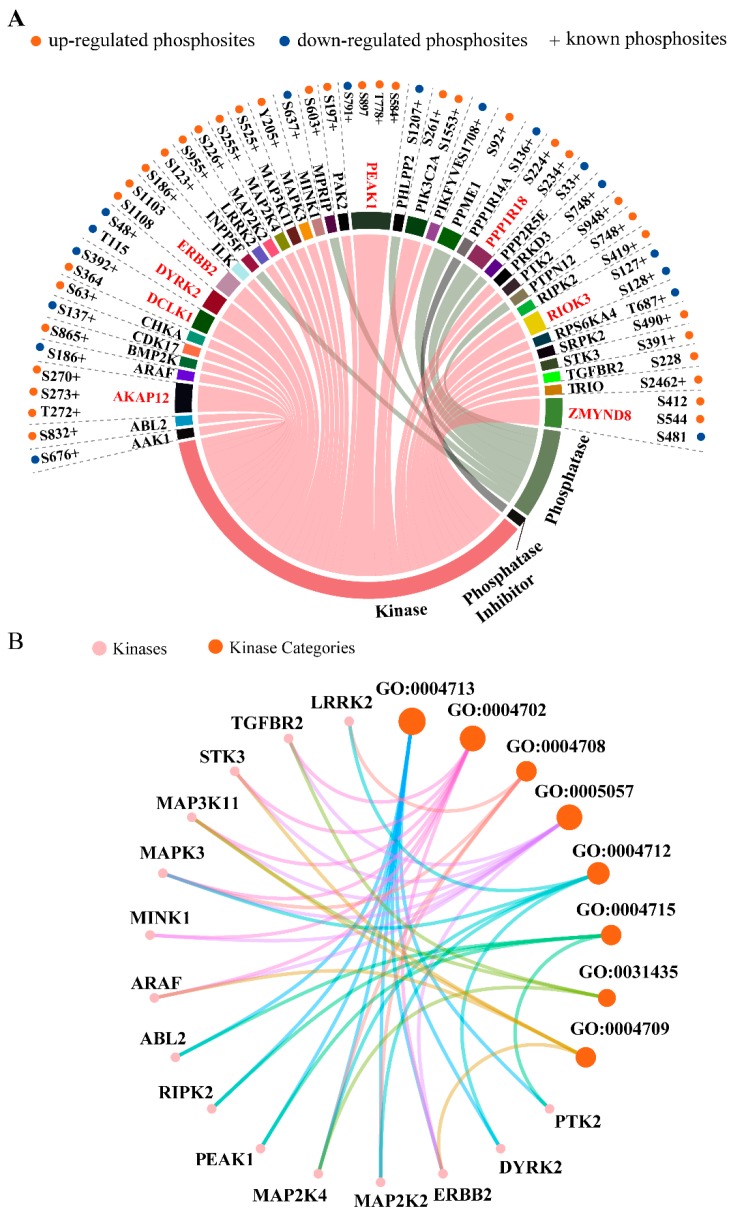
Phosphorylation in kinases, phosphatases and phosphatase inhibitors regulated by SPRY. (**A**) The chord diagram shows the detailed distributions of the phosphosites on the identified kinases, phosphatases and phosphatase inhibitors. In the chord diagram, 17 of the 52 sites are down-regulated (labeled with blue dots) and eight of the 39 proteins have more than one phosphorylation site (marked in red). Orange dots represent up-regulated phosphosites, and blue dots are down-regulated phosphosites. In addition, previously reported phosphosites are labeled with “+”; (**B**) The net plot shows the top eight kinase function categories containing 15 kinases in (**A**). Kinase categories are shown in orange, and kinases are in pink. The matched descriptions of GO terms IDs are in Table S4.

In order to derive an overall protein classification of the up-/down-regulated phosphoproteins, we performed a functional categories analysis using Database for Annotation, Visualization and Integrated Discovery (DAVID)-based functional annotation (Figure 2D). The results showed that most of the proteins (370 of 415, ~89.1%) are phosphoproteins. Among those 370 phosphoproteins, we identified seven phosphatases that are: INPP5F (S123), MPRIP (S603), PHLPP2 (S1242), PPME1 (S92), PPP1R18 (S234), PPP2R5E (S33) and PTPN12 (S748) (Figure 3A). Of the seven phosphatases, two proteins, PHLPP2 and PPP2R5E, with known sites S1207 and S33, were decreased (Figure 3A). PHLPP2 was reported as a phosphatase that terminates growth factor signaling by dephosphorylating AKT [46,47]. Only one phosphatase inhibitor PPP1R14A was discovered with down-regulated phosphorylation of the known site S136 in our dataset. Of all the regulated phosphosites, a total of 43 sites (~7.8%) on 31 kinases was identified (Figure 2D and Figure 3A). The chord diagram displays the detailed distributions of the phosphosites on the identified kinase, phosphatase and phosphatase inhibitor (Figure 3A). As shown in the chord diagram, 17 of the 52 sites are down-regulated, and eight of the 39 proteins have more than one phosphorylation site. 

To further understand the specific function of each kinase, we employed molecular function analysis of the 31 kinases. The net plot shows the top eight function categories containing 15 kinases (Figure 3B). There are eight kinases with identified phosphosites involved in protein tyrosine kinase activity (GO:0004713): ABL2 (S832), RIPK2 (S419), PEAK1 (S584, T778, S897 and S791), MAP2K4 (S255), MAP2K2 (S226), ERBB2 (S1103 and S1108), DYRK2 (S48 and T115) and PTK2 (S948). Two phosphorylation sites S48 (known site) and T115 (unknown site) on DYRK2 were both decreased. DYRK2 is reported as a serine/threonine-protein kinase involved in the control of the cell cycle, cell proliferation, apoptosis, cytoskeletal organization and neurite outgrowth [48]. Interestingly, we identified three up-regulated phosphosites on the two well-documented proteins ERBB2 and TGFBR2: S1103 and S1108 of ERBB2, and S2208 of TGFBR2, that were previously unknown. Both proteins are important signal transducers downstream of receptor signaling with serine/threonine/tyrosine kinase activity. Our data indicate the possibility of a new approach by which SPRY modulates downstream receptor signaling cascades.

### 2.4. Phosphorylation in Established Protein Complexes Regulated by SPRY in Mammary Stromal Fibroblasts

**Figure 4:**
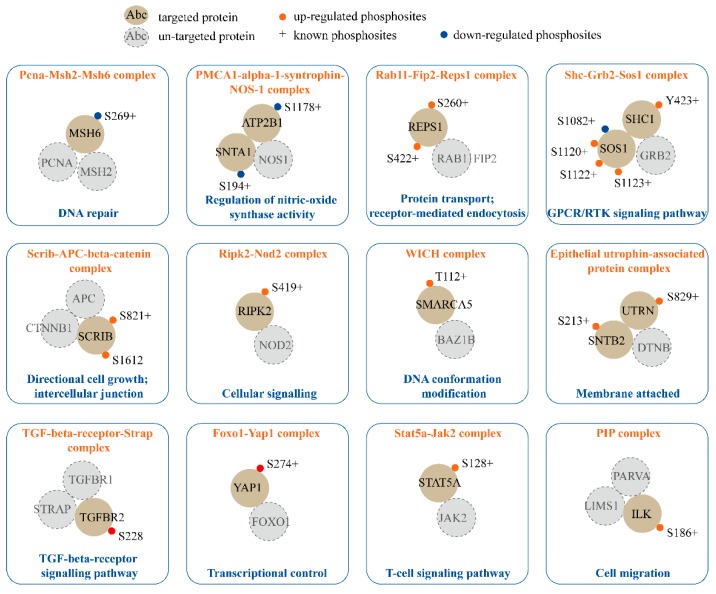
SPRY regulates phosphorylation in the established protein complexes variably. Twelve complexes participating in different cellular processes are selected and annotated according to GO-based descriptions. The 12 complexes encompass 15 proteins with 20 phosphosites. Khaki circles indicate targeted proteins in the phosphoproteome. Gray circles indicate proteins that are not targeted. Orange dots represent up-regulated phosphosites and down-regulated phosphosites are marked with blue dots. Previously reported phosphosites are labeled with “+”. WICH complex: WSTF-ISWI chromatin remodeling complex; PIP complex: PINCH-1/2/ILK/α-parvin complex.

Phosphorylation mediates protein complex formation to regulate signal effector activities and signaling transduction in various biological processes [49,50]. In this study, we discovered up to 100 established protein complexes (Table S5) containing components in the SPRY-deficiency-based phosphoproteomic dataset by utilizing the CORUM database [51]. 12 complexes encompassing 15 proteins with 20 phosphosites in our dataset are demonstrated and annotated according to gene-ontology-based descriptions (Figure 4, and Table S5). Both the Shc-Grb2-Sos1 complex and TGF-beta-receptor-Strap complex were included, which function in the GPCR/RTK signaling pathway and the TGF-beta-receptor signaling pathway, respectively. Interestingly, we found that the Stat5a-Jak2 complex, which is involved in T cell differentiation [52], had an increased phosphorylation site at S128 on STAT5A. Recently, Shehata et al. had also reported that SPRY1 and SPRY2 regulate CD8+ T cell memory development by influencing the mTOR–Akt–FoxO signaling axis, but the detailed mechanism is still unclear [18]. Shehata et al.’s work is the first report to reveal the connections between SPRY and T cell signaling [18,38], our data may provide new insights into this filed.

Furthermore, previously unknown relationships between SPRY and the phosphorylation of several protein complexes, such as the Pcna-Msh2-Msh6 complex in DNA repair, the PMCA1-alpha-1-syntrophin-NOS-1 complex in the regulation of nitric-oxide synthase activity, and the WSTF-ISWI chromatin remodeling (WICH) complex in DNA conformation modification, are also included in our results. In summary, these results indicate that SPRY-mediated protein complexes are involved in a variety of biological functions, including protein transport, intercellular junction, transcriptional control, cell migration and signaling pathway regulation (Figure 4, Table S5).

### 2.5. An Receptor Tyrosine Kinase (RTK)-Centric Signaling Network Regulated by SPRY in Mammary Stromal Fibroblasts

Pathways are fundamental delivery channels for biochemical signal transduction. When signaling pathways interact with each other, networks are formed which allow the cellular responses to be coordinated, typically by combining signaling events [53].

**Figure 5:**
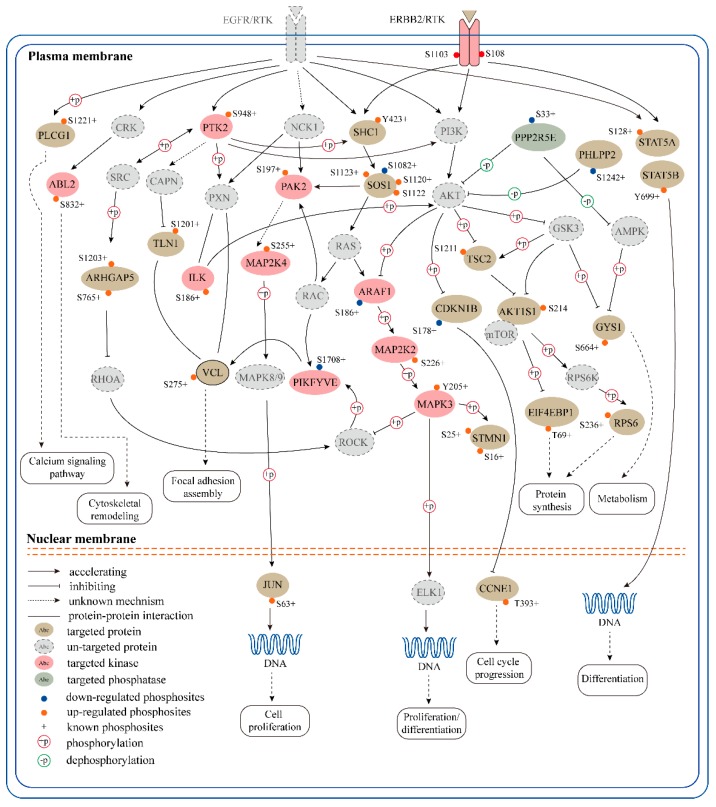
SPRY-altered signaling network profiles in mammary stromal fibroblasts. A phosphorylation network centered on receptor tyrosine kinase (RTK) signaling cascades was reconstructed according to KSR and KEGG pathway enrichment. The biological processes underlying the pathway network, such as cytoskeleton remodeling, cell proliferation and cell cycle progression, are shown in the schematic diagram. Khaki ellipses indicate targeted proteins, while gray ellipses indicate un-targeted proteins in this study. Kinases and phosphatase inhibitors are shown in pink and gray-green ellipses, respectively. Orange dots represent up-regulated phosphosites and down-regulated phosphosites are marked with blue dots and previously reported phosphosites are labeled with “+”. The different line types indicate different regulation mechanisms between two proteins. Phosphorylating and dephosphorylating relationships are also illustrated in the diagram with red and green circles, respectively.

To reconstitute signaling pathways perturbed by SPRY, we reconstructed the phosphorylation network centered on RTK signaling cascades according to two approaches: Established kinase/phosphatase-substrate relationships and KEGG pathway enrichment analysis of phosphorylation proteins regulated by SPRY. As shown in Figure 5, we grouped ErbB signaling, EGFR signaling, MAPK signaling, PI3K-Akt signaling and the focal adhesion signaling pathway by their shared molecules or interaction relationships. Cellular processes related to metabolism, cytoskeletal remodeling, protein synthesis and cell proliferation/differentiation were specified according to the signaling branches that perform specific functions. The reconstituted pathway leads to a comprehensive overview of the SPRY-regulated RTK signaling network, and provides a resource system in the exploration of the roles of SPRY and RTK signaling pathways in mammary stromal fibroblasts.

## 3. Discussion

Fibroblasts, as the most prominent stromal components, are essential in regulating normal development and tumor progression. In particular, cancer-associated fibroblasts (CAFs) have been widely described to relate to drug resistance [54,55] and to diminish anti-tumor immunity [56,57,58,59,60]. In breast cancer, the abundance of stromal fibroblasts has been associated with aggressive adenocarcinomas and cancer recurrence [61,62]. It has been reported that during breast carcinogenesis, activated CAFs have active roles in the initiation, progression and metastasis of tumor [10]. The activation of CAFs can be induced by cytokines secreted by cancer cells, including TGF-β and several RTK signaling ligands such as EGF, PDGF and FGF2 [63]. Meanwhile, cell-cell contact through intercellular adhesion molecules can also enable fibroblasts activation [64].

Elevated levels of RTK signaling are found to be highly associated with increased breast cancer deterioration and decreased survival potential [65]. Here, by employing two GEO datasets (GSE88715 [66] and GSE20086 [67]) to perform gene set enrichment analysis (GSEA) in breast CAFs, we found that several RTK downstream signaling processes, such as RAS signaling, MAPK signaling, and PI3K-AKT signaling were significantly enriched. This indicates elevated RTK signaling activity in breast cancer stromal fibroblasts (Figure S1A,B). Comparing with breast CAFs, similar repercussions were observed with deletions of SPRY1, 2 and 4 in normal mammary fibroblasts, which suggested that SPRY loss may promote CAF-like behaviors (Figure S2C,D). By quantifying the large-scale phosphoproteome in *SpryKO* mouse mammary stromal fibroblasts, we showed that several RTK signaling pathways, including the ErbB signaling pathway, insulin signaling pathway and VEGF signaling pathways, were up-regulated, as well as the focal adhesion pathway and mTOR signaling pathway. Protein functional categories analysis uncovered the phosphorylation status of 31 kinases, 7 phosphatases, and one phosphatase inhibitor that was significantly changed. Over 100 established protein complexes were identified that are involved in broad aspects of the cellular process. Based on the above results, we reconstructed a system-wide phosphorylation network centered on RTK signaling to reconstitute signaling cascades that are perturbed by SPRY loss. 

For the first time, this study revealed systemic phosphorylation networks regulated by SPRY in murine mammary gland stromal fibroblasts. While fibroblasts are the most prominent component of the mammary stromal ecosystem, characterizing their function in regulating normal mammary gland development and cancer progression is still far from complete. Recently, single-cell-based transcriptomics and proteomics studies have revealed the heterogeneity and immunosuppression function of breast CAFs [68,69]. These findings support that CAFs can be a potential target for breast cancer therapy. By utilizing a large-scale quantitative phosphoproteomics analysis, we profiled a global phosphorylation system containing molecules in SPRY-regulated RTK signaling and other signaling pathways. However, the limitation of this study is that we have not verified the phosphorylation residues on the identified proteins. 

However, one of the increased phosphorylation sites, Y205 on MAPK3/ERK1, is consistent with our previous findings in Spry1-deleted mouse mammary stromal fibroblasts. Despite the limitations of this study, we believe our results will provide an important resource for future studies of normal mammary fibroblasts and CAFs in regulating mammary gland development and tumorigenesis. Taken together, by employing an in-depth phosphoproteomics investigation of the SPRY-responsive signaling networks, this study improves our understandings of the molecular mechanisms regulated by SPRY in mammary stromal fibroblasts.

## 4. Materials and Methods

### 4.1. Mice and Cells

Mice carrying Sprouty protein 1 (Spry1) ^flox/flox^, 2 ^flox/flox^, and 4 ^flox/flox^ were provided by Gail R. Martin, Department of Anatomy and Program in Developmental Biology, School of Medicine, University of California at San Francisco, San Francisco, California. Mice were genotyped as described in the publications establishing the mouse lines [70,71,72]. All animal experiments were conducted according to guidelines set by the Institutional Animal Care and Use Committee (IACUC# 2015SHT0006) of Shanghai Tech University (01December 2015).

The primary normal fibroblasts from mice carrying Spry1 ^flox/flox^, 2 ^flox/flox^, and 4 ^flox/flox^ were used for the phsphoproteomic experiment. Adenovirus-GFP-infected cells were used as a control group (referred to as control fibroblasts), and adenovirus-Cre-GFP-infected cells were used as an experimental group (referred to as SpryKO fibroblasts).

### 4.2. Isolation of Primary Mouse Mammary Stromal Fibroblasts

Primary mouse mammary fibroblasts were isolated, as previously described [6]. In brief, mouse mammary glands were minced until the tissue relaxes, and minced glands were digested in collagenase solution (with 0.2 g/mL collagenase (Merck KGaA, Darmstadt, Germany), 0.2 g/mL trypsin (Thermo Fisher, MA, USA), 5 µg/mL insulin, 50 µg/mL gentamicin, 5% fetal bovine serum (FBS) (*v*/*v*) (Gemini, CA, USA), and DMED/F12 (Corning, NY, USA) for 30 min. Samples were centrifuged, washed twice, and treated with DNase I (Thermo Fisher, MA, USA). Then samples were subjected to 4–5 times of differential centrifugation (a short-pulse centrifugation at 450× *g*), during which supernatant was collected and centrifuged, and cell pellets were resuspended in Fibroblast medium (DMEM with 10% (*v*/*v*) FBS, 1× ITS (Merck KGaA, Darmstadt, Germany), 1× penicillin and streptomycin (Thermo Fisher, MA, USA)) and seeded on a Petri dish. After 30 min, the medium was aspirated, and cells were washed twice with cold PBS and fresh Fibroblast medium was added. Cells were cultured in the incubator at 37 °C with 5% CO2 for later use.

### 4.3. Adenovirus Infection and Fluorescence-Activated Cell Sorting (FACS)

Cells were trypsinized and centrifuged at 650× *g* for 2–3 min. Then the cells were resuspended in the fibroblast medium and infected with Adenovirus-Cre-GFP (green fluorescent protein) or Adenovirus-GFP overnight at a multiplicity of infection of ~25 particles per cell. After infection, fibroblasts were washed twice with cold PBS and resuspended in FACS buffer (Hanks solution with 2% FBS). Cell sorting was conducted using an Aria system (BD, NJ, USA). Cells were collected into the round bottom polystyrene test tubes (Corning, NY, USA) with fresh Fibroblast medium for use.

### 4.4. Cell lysate Preparation, Protein Digestion, and Peptide Desalting

About one million cells were centrifuged and disrupted with 100 µL lysis buffer (with 50 mM triethylammonium bicarbonate (TEAB), 8 M urea, the protease inhibitor (Roche, Basel, Switzerland), and 1 mM dithiothreitol (DTT)) in an ultrasonic system on ice for 30 min. Protein quantitation was performed using the BCA protein assay kit (Thermo Fisher, MA, USA). A final concentration of 4 mM DTT was added to the protein solution, which was then incubated at 37 °C for 2 h. 

Iodoacetamide (IAM) was added to the protein solution at a final concentration of 20 mM and the tubes were placed in the dark at room temperature for 45 min. Then we added 50 mM TEAB to the tubes to dilute the urea concentration to 1 M. Trypsin was added at a ratio of 1:50 (*v*/*v*) to digest the protein at 37 °C overnight. Then we add 0.4% trifluoroacetic acid (TFA) and adjust the pH to 2–4 (more acidic). The protein digest was centrifuged at 10,000× *g* for 10 min. Peptide desalting was done by using Sep-Pak devices (Waters, MA, USA). The Sep-Pak C18 columns were used according to the user manual. After desalting, the peptides were dried in the SpeedVac Vacuum Concentrator (Thermo Fisher, MA, USA) and then stored at −80 °C for later use.

### 4.5. Tandem Mass Tag (TMT) Labeling

The TMT10plex Mass Tag Labeling Kit (Thermo Fisher, MA, USA) was used for peptide labeling according to the standard protocol from the user guide. Briefly, the dried peptides were separately dissolved in 100 µL of 50 mM TEAB. Before use, immediately equilibrate the Tandem Mass Tag (TMT) Label Reagents to room temperature. Add 43 µL of anhydrous acetonitrile to each tube. Allow the reagent to dissolve for 5 min with occasional vorticing, and then briefly centrifuge the tube to gather the solution. Carefully transfer the 43 µL of the TMT Label Reagent to each 100 µL sample and incubate the reaction for 1h at room temperature. Add 8 µL of 5% hydroxylamine to the sample and incubate for 15 min to quench the reaction. Combine samples in a new tube and speedvac to dryness to remove TEAB from the labeled peptide sample. Labeled peptides desalting was done by using the Sep-Pak devices (Waters) as described above. After desalination, the peptides were dried and stored at −80 °C.

### 4.6. Phosphopeptide Enrichment

The Pierce TiO2 Phosphopeptide Enrichment Kit (Thermo Fisher, MA, USA) was used for phosphopeptide enrichment according to the standard protocol from the user guide. Briefly, the Centrifuge Column Adaptor was placed into a collection tube, and a TiO_2_ Spin Tip was inserted into the adaptor. Then 20 µL Buffer A (with 2% TFA and acetonitrile) and Buffer B (Buffer A and 50% lactic acid) were sequentially added, followed by centrifugation at 3000× *g* for 2 min, respectively. Peptide samples were suspended in 100 µL of Buffer B and applied to the spin tip to centrifuge at 1000× *g* for 10 min. Reapply samples to the spin tip and centrifuge at 1000× *g* for 10 min, 5–6 times. Then wash the columns once with 20 µL of Buffer B and twice with 20 µL of Buffer A. Finally, the samples were eluted twice with 20 µL of elution buffer (5% ammonia and 40% acetonitrile), centrifuged at 1000× *g* for 10 min, combined and drained, and placed at −80 °C for later use.

### 4.7. Mass Spectrometry and Data Processing

Peptides were separated and analyzed by using an Easy-nLC 1000 system coupled to a Q Exactive HF (both from Thermo Scientific, MA, USA). About 2 µg of peptides were separated in an EASY-Spay column (75 µm × 50 cm) packed with C18 AQ (2 µm, 100Å) at a flow rate of 250 nL/min. Mobile phase A (0.1% formic acid in 2% ACN) and mobile phase B (0.1% formic acid in 98% ACN) were used to establish a 120 min gradient comprised of 1 min of 5% B, 105 min of 5–28% B, 2 min of 28–38% B, 1 min of 38–90% B and 10 min of 90% B. Peptides were then ionized by electrospray at 2.3 kV. A full MS spectrum (375–1400 *m/z* range) was acquired at a maximum ion accumulation time of 50 ms and a resolution of 120,000 at *m/z* 200. Dynamic exclusion was set to 30 s. Resolution (higher-energy collisional Dissociation) for HCD MS/MS spectra was set to 60,000 at *m/z* 200. The automatic gain control (AGC) setting of MS and MS^2^ were set at 3E6 and 1E5, respectively. The 20 most intense ions above the 1.7E4 count threshold were selected to be fragmented by HCD with a maximum ion accumulation time of 120 ms. The isolation width of 1.2 *m/z* units was used for MS^2^. Single and unassigned charged ions were excluded from MS/MS. For HCD, the normalized collision energy was set to 30%. The underfill ratio was defined as 1%. 

The raw data were processed and searched with MaxQuant 1.5.4.1 with MS tolerance of 4.5 p.p.m., and MS/MS tolerance of 20 p.p.m. The UniProt mouse protein database (release 2016_07, 49,838 sequences), and database for proteomics contaminants from MaxQuant were used for database searches. Reversed database searches were used to evaluate the false discovery rate (FDR) of peptide and protein identifications. Two missed cleavage sites of trypsin were allowed. Oxidation (M), acetyl (protein *N*-term), deamidation (NQ) and Phospho (STY) were set as variable modifications. The FDR of both peptide identification and protein identification was set to be 1%. The options of “Second peptides”, “Match between runs”, and “Dependent peptides” were enabled. The “phospho (STY)” output file from the MaxQuant was processed by “Perseus 1.6.1.3” for downstream data analysis. Data processing by Perseus was configured as follows: Reverse and potential contaminant hits were removed, and the minimum localization probability of 0.75 was set to accept phosphosites for further analysis. For the proteomics and phosphoproteomics data, median normalization was performed at the phosphopeptide level accordingly, to ensure all medians were 0 [73,74].

### 4.8. Bioinformatics Analysis, Statistics and Data Visualization

For differential expression analysis, the limma-based approach was used in R 3.5.0. The data were log_2_ transformed and centered, and the statistical significance of the biological duplicates of *SpryKO* and control was tested using a modified t-test in limma 3.40.0. Phosphosites with fold change > 1.5 and *p*-value < 0.05 were defined as up-regulated sites and those with foldchange < 0.67 and *p*-value < 0.05 were defined as down-regulated sites. Functional enrichment for the up-/down-regulated phosphoproteins was obtained as GO terms corresponding to biological process (BP), cellular component (CC) and molecular function (MF) based on the clusterProfiler software [75]. Only those categories with *p*-adjust less than 0.05 were considered to be reliable. The top 15 GO molecular function categories in the up-regulated phosphoproteins were visualized as a heat plot. Kyoto Encyclopedia of Genes and Genomes (KEGG) pathway enrichment analysis was conducted using clusterProfiler, and the top 18 enriched terms (*p*-adjust < 0.05) of the up-regulated phosphoproteins were also visualized as a heat plot. The relationships between enriched terms were analyzed using an online tool “Metascape”, and visualized using Cytoscape 3.7.1. The functional categories analysis of all regulated phosphoproteins was performed using the Database for Annotation, Visualization and Integrated Discovery (DAVID) v6.8-based functional annotation in terms of “UP_KEYWORDS”. The known phosphosites and kinase-substrate relations (KSR) were identified in Perseus by employing “Kinase Substrate Dataset” and “Phosphorylation Site Dataset” from PhosphoSitePlus. Established protein complexes containing regulated phosphoproteins were identified by the comprehensive resource of mammalian protein complexes (CORUM) database. The regulatory network was constructed based on KSR, KEGG pathway and published literature. Statistical significance was assessed on the basis of *p*-values calculated via Student’s t-test (two-tailed, * *p* < 0.05, ** *p* < 0.01, *** *p* < 0.001, **** *p* < 0.0001) in R 3.5.0. The data related to breast CAFs were obtained from GEO database. GSEA was conducted using the enrichplot package 1.2.0. All the data visualizations were performed in R 3.5.0 and Adobe Illustrator.

## Figures and Tables

**Table 1:** List of known Sprouty protein (SPRY) functions in biological processes.

SPRY1	SPRY2	SPRY3	SPRY4
bud elongation involved in lung branching;	branching morphogenesis of an epithelial tube;	axon development;	multicellular organism development;
EMT involved in cardiac fibroblast development;	bud elongation involved in lung branching;	multicellular organism development;	negative regulation of ERK1 and ERK2 cascade;
metanephros development;	cell fate commitment;	regulation of signal transduction.	regulation of signal transduction.
multicellular organism development;	cellular response to leukemia inhibitory factor		
negative regulation of cell proliferation;	cellular response to VEGF stimulus;		
negative regulation of EGF receptor signaling pathway;	establishment of mitotic spindle orientation;		
negative regulation of ERK1 and ERK2 cascade;	inner ear morphogenesis;		
negative regulation of FGF receptor signaling pathway;	lung development;		
negative regulation of GTPase activity;	lung growth;		
negative regulation of MAP kinase activity;	lung morphogenesis;		
negative regulation of neurotrophin TRK receptor signaling pathway;	multicellular organism development;		
negative regulation of Ras protein signal transduction;	negative regulation of angiogenesis;		
organ induction;	negative regulation of apoptotic process;		
regulation of signal transduction;	negative regulation of cell projection organization;		
ureteric bud development.	negative regulation of cell proliferation;		
	negative regulation of EGF receptor signaling pathway;		
	negative regulation of ERK1 and ERK2 cascade;		
	negative regulation of FGF receptor signaling pathway;		
	negative regulation of GTPase activity;		
	negative regulation of MAP kinase activity;		
	negative regulation of peptidyl;		
	negative regulation of Ras protein signal transduction;		
	negative regulation of VEGF signaling pathway;		
	positive regulation of cell migration;		
	positive regulation of ERK1 and ERK2 cascade;		
	positive regulation of gene expression;		
	positive regulation of peptidyl-serine phosphorylation;		
	positive regulation of protein kinase B signaling;		
	positive regulation of protein serine/threonine kinase activity;		
	regulation of cell differentiation;		
	regulation of cell proliferation;		
	regulation of signal transduction;		
	respiratory system development;		
	sensory perception of sound.		

Data were collected from the National Center for Biotechnology Information (NCBI) Database, the UniProt database, and the GeneCards Database.

## Supplementary Materials

Supplementary materials can be found at https://www.mdpi.com/1422-0067/20/21/5400/s1.

## Abbreviations

AAK1	AP2 associated kinase 1
ABL2	v-abl Abelson murine leukemia viral oncogene 2 (arg, Abelson-related gene)
Ad	adenovirus
AGC	automatic gain control
AKAP12	A kinase (PRKA) anchor protein (gravin) 12
AKT1S1	AKT1 substrate 1 (proline-rich)
ARAF	Araf proto-oncogene, serine/threonine kinase
ARHGAP42	Rho GTPase activating protein 42
ARHGAP5	Rho GTPase activating protein 5
ARHGEF12	Rho guanine nucleotide exchange factor (GEF) 12
ARHGEF40	Rho guanine nucleotide exchange factor (GEF) 40
ATP2B1	ATPase, Ca++ transporting, plasma membrane 1
BMP2K	BMP2 inducible kinase
BP	biological process
CAFs	cancer-associated fibroblasts
CC	cellular component
CCNE1	cyclin E1
CDK17	cyclin-dependent kinase 17
CDKN1B	cyclin-dependent kinase inhibitor 1B
CHKA	choline kinase alpha
CORUM	the comprehensive resource of mammalian protein complexes
Cre	cyclization recombination enzyme
DAVID	the database for annotation, visualization and integrated discovery
DCLK1	doublecortin-like kinase 1
DOCK9	dedicator of cytokinesis 9
DTT	Dithiothreitol
DYRK2	dual-specificity tyrosine-(Y)-phosphorylation regulated kinase 2
ECM	extracellular matrix
EGF	epidermal growth factor
EGFR	epidermal growth factor receptor
EIF4EBP1	eukaryotic translation initiation factor 4E binding protein 1
ERBB2	erb-b2 receptor tyrosine kinase 2
ERK	extracellular regulated kinase
FACS	fluorescence activated cell sorting
FARP1	FERM, RhoGEF (Arhgef) and pleckstrin domain protein 1 (chondrocyte-derived)
FBS	fetal bovine serum
FDR	false discovery rate
FGD1	FYVE, RhoGEF and PH domain containing 1
FGF	fibroblast growth factor
GDP	guanosine diphosphate
GEO	gene expression omnibus
GFP	green fluorescent protein
GO	Gene Ontology
GPCR	G protein-coupled receptors
GSEA	gene Set Enrichment Analysis
GTP	guanosine triphosphate
GYS1	glycogen synthase 1, muscle
HCD	higher-energy collisional dissociation
IAM	Iodoacetamide
ILK	integrin linked kinase
INPP5F	inositol polyphosphate-5-phosphatase F
ITS	insulin transferrin selenium
JUN	jun proto-oncogene
KEGG	kyoto encyclopedia of genes and genomes
KLHL32	kelch-like 32
KSR	kinase-substrate relationships
LC-MS/MS	large-scale liquid chromatography tandem mass spectrometry
LRRK2	leucine-rich repeat kinase 2
MAP1B	microtubule-associated protein 1B
MAP2K2	mitogen-activated protein kinase kinase 2
MAP2K4	mitogen-activated protein kinase kinase 4
MAP3K11	mitogen-activated protein kinase kinase kinase 11
MAPK	mitogen-activated protein kinase
MAPK3	mitogen-activated protein kinase 3
MF	molecular function
MINK1	misshapen-like kinase 1 (zebrafish)
MMPs	matrix metalloproteinases
MPRIP	myosin phosphatase Rho interacting protein
MS	mass spectrometry
MSH6	mutS homolog 6
mTOR	mammalian target of rapamycin
MYOT	myotilin
PAK2	p21 (RAC1) activated kinase 2
PBS	phosphate buffered saline
PDGF	platelet-derived growth factor
PEAK1	pseudopodium-enriched atypical kinase 1
PHLPP2	PH domain and leucine rich repeat protein phosphatase 2
PIK3C2A	phosphatidylinositol-4-phosphate 3-kinase catalytic subunit type 2 alpha
PIKFYVE	phosphoinositide kinase, FYVE type zinc finger containing
PIP complex	PINCH-1/2/ILK/α-parvin complex
PLCG1	phospholipase C, gamma 1
PPME1	protein phosphatase methylesterase 1
PPP1R14A	protein phosphatase 1, regulatory inhibitor subunit 14A
PPP1R18	protein phosphatase 1, regulatory subunit 18
PPP2R5E	protein phosphatase 2, regulatory subunit B’, epsilon
PRKD3	protein kinase D3
PTK2	PTK2 protein tyrosine kinase 2
PTPN12	protein tyrosine phosphatase, non-receptor type 12
RAB11FIP5	RAB11 family interacting protein 5 (class I)
RAB3IL1	RAB3A interacting protein (rabin3)-like 1
RAB3IP	RAB3A interacting protein
REPS1	RalBP1 associated Eps domain containing protein
RIOK3	RIO kinase 3
RIPK2	receptor (TNFRSF)-interacting serine-threonine kinase 2
RPS6	ribosomal protein S6
RPS6KA4	ribosomal protein S6 kinase, polypeptide 4
RTK	receptor tyrosine kinase
S	serine
T	Threonine
SCRIB	scribbled planar cell polarity
SHC1	src homology 2 domain-containing transforming protein C1
SMARCA5	SWI/SNF related, matrix associated, actin dependent regulator of chromatin, subfamily a, member 5
SMCR8	Smith-Magenis syndrome chromosome region, candidate 8 homolog (human)
SNTA1	syntrophin, acidic 1
SNTB2	syntrophin, basic 2
SOS1	SOS Ras/Rac guanine nucleotide exchange factor 1
SPRY	Sprouty
SpryKO	Spry1, 2 and 4 triple knock out
SRPK2	serine/arginine-rich protein specific kinase 2
STAT5A	signal transducer and activator of transcription 5A
STAT5B	signal transducer and activator of transcription 5B
STK3	serine/threonine kinase 3
STMN1	stathmin 1
TBC1D10A	TBC1 domain family, member 10a
TEAB	triethyl ammonium bicarbonate
TFA	trifluoroacetic acid
TGFBR2	transforming growth factor, beta receptor II
TLN1	talin 1
TMT	tandem mass tag
TRIO	triple functional domain (PTPRF interacting)
TSC2	TSC complex subunit 2
UTRN	Utrophin
VCL	Vinculin
VEGF	vascular endothelial growth factor
WICH complex	WSTF-ISWI chromatin remodeling complex
Y	Tyrosine
YAP1	yes-associated protein 1
ZBTB11	zinc finger and BTB domain containing 11
ZMYND8	zinc finger, MYND-type containing 8

## References

[1] Lane S.W., Williams D.A., Watt F.M. (2014). Modulating the stem cell niche for tissue regeneration. Nat. Biotechnol..

[2] Quail D.F., Joyce J.A. (2013). Microenvironmental regulation of tumor progression and metastasis. Nat. Med..

[3] Rozario T., DeSimone D.W. (2010). The extracellular matrix in development and morphogenesis: A dynamic view. Dev. Biol..

[4] Chen X., Song E. (2019). Turning foes to friends: Targeting cancer-associated fibroblasts. Nat. Rev. Drug Discov..

[5] Driskell R.R., Lichtenberger B.M., Hoste E., Kretzschmar K., Simons B.D., Charalambous M., Ferron S.R., Herault Y., Pavlovic G., Ferguson-Smith A.C. (2013). Distinct fibroblast lineages determine dermal architecture in skin development and repair. Nature.

[6] Koledova Z., Zhang X., Streuli C., Clarke R.B., Klein O.D., Werb Z., Lu P. (2016). SPRY1 regulates mammary epithelial morphogenesis by modulating EGFR-dependent stromal paracrine signaling and ECM remodeling. Proc. Natl. Acad. Sci. USA.

[7] Bhowmick N.A., Neilson E.G., Moses H.L. (2004). Stromal fibroblasts in cancer initiation and progression. Nature.

[8] Kim J.B., Stein R., O’Hare M.J. (2005). Tumour-stromal interactions in breast cancer: The role of stroma in tumourigenesis. Tumour Biol..

[9] Schor S.L., Schor A.M. (2001). Phenotypic and genetic alterations in mammary stroma: Implications for tumour progression. Breast Cancer Res..

[10] Aboussekhra A. (2011). Role of cancer-associated fibroblasts in breast cancer development and prognosis. Int. J. Dev. Biol..

[11] Hanahan D., Weinberg R.A. (2011). Hallmarks of cancer: The next generation. Cell.

[12] Zhang J., Liu J. (2013). Tumor stroma as targets for cancer therapy. Pharmacol. Ther..

[13] Brechbuhl H.M., Finlay-Schultz J., Yamamoto T.M., Gillen A.E., Cittelly D.M., Tan A.C., Sams S.B., Pillai M.M., Elias A.D., Robinson W.A. (2017). Fibroblast Subtypes Regulate Responsiveness of Luminal Breast Cancer to Estrogen. Clin. Cancer Res..

[14] Marusyk A., Tabassum D.P., Janiszewska M., Place A.E., Trinh A., Rozhok A.I., Pyne S., Guerriero J.L., Shu S., Ekram M. (2016). Spatial Proximity to Fibroblasts Impacts Molecular Features and Therapeutic Sensitivity of Breast Cancer Cells Influencing Clinical Outcomes. Cancer Res..

[15] Fantl W.J., Johnson D.E., Williams L.T. (1993). Signalling by receptor tyrosine kinases. Annu. Rev. Biochem..

[16] Casaletto J.B., McClatchey A.I. (2012). Spatial regulation of receptor tyrosine kinases in development and cancer. Nat. Rev. Cancer.

[17] Ching S.T., Cunha G.R., Baskin L.S., Basson M.A., Klein O.D. (2014). Coordinated activity of Spry1 and Spry2 is required for normal development of the external genitalia. Dev. Biol..

[18] Shehata H.M., Khan S., Chen E., Fields P.E., Flavell R.A., Sanjabi S. (2018). Lack of Sprouty 1 and 2 enhances survival of effector CD8(+) T cells and yields more protective memory cells. Proc. Natl. Acad. Sci. USA.

[19] Dikic I., Giordano S. (2003). Negative receptor signalling. Curr. Opin. Cell Biol..

[20] Qian B.Z., Zhang H., Li J., He T., Yeo E.J., Soong D.Y., Carragher N.O., Munro A., Chang A., Bresnick A.R. (2015). FLT1 signaling in metastasis-associated macrophages activates an inflammatory signature that promotes breast cancer metastasis. J. Exp. Med..

[21] Butti R., Das S., Gunasekaran V.P., Yadav A.S., Kumar D., Kundu G.C. (2018). Receptor tyrosine kinases (RTKs) in breast cancer: Signaling, therapeutic implications and challenges. Mol. Cancer.

[22] Sorlie T., Perou C.M., Tibshirani R., Aas T., Geisler S., Johnsen H., Hastie T., Eisen M.B., van de Rijn M., Jeffrey S.S. (2001). Gene expression patterns of breast carcinomas distinguish tumor subclasses with clinical implications. Proc. Natl. Acad. Sci. USA.

[23] Faratian D., Sims A.H., Mullen P., Kay C., Um I., Langdon S.P., Harrison D.J. (2011). Sprouty 2 is an independent prognostic factor in breast cancer and may be useful in stratifying patients for trastuzumab therapy. PLoS ONE.

[24] Ley R., Balmanno K., Hadfield K., Weston C., Cook S.J. (2003). Activation of the ERK1/2 signaling pathway promotes phosphorylation and proteasome-dependent degradation of the BH3-only protein, Bim. J. Biol. Chem..

[25] Beltran L., Chaussade C., Vanhaesebroeck B., Cutillas P.R. (2011). Calpain interacts with class IA phosphoinositide 3-kinases regulating their stability and signaling activity. Proc. Natl. Acad. Sci. USA.

[26] Olsen J.V., Blagoev B., Gnad F., Macek B., Kumar C., Mortensen P., Mann M. (2006). Global, in vivo, and site-specific phosphorylation dynamics in signaling networks. Cell.

[27] Yi T., Zhai B., Yu Y., Kiyotsugu Y., Raschle T., Etzkorn M., Seo H.C., Nagiec M., Luna R.E., Reinherz E.L. (2014). Quantitative phosphoproteomic analysis reveals system-wide signaling pathways downstream of SDF-1/CXCR4 in breast cancer stem cells. Proc. Natl. Acad. Sci. USA.

[28] Matheron L., van den Toorn H., Heck A.J., Mohammed S. (2014). Characterization of biases in phosphopeptide enrichment by Ti(4+)-immobilized metal affinity chromatography and TiO2 using a massive synthetic library and human cell digests. Anal. Chem..

[29] Huttlin E.L., Ting L., Bruckner R.J., Gebreab F., Gygi M.P., Szpyt J., Tam S., Zarraga G., Colby G., Baltier K. (2015). The BioPlex Network: A Systematic Exploration of the Human Interactome. Cell.

[30] Kronke J., Fink E.C., Hollenbach P.W., MacBeth K.J., Hurst S.N., Udeshi N.D., Chamberlain P.P., Mani D.R., Man H.W., Gandhi A.K. (2015). Lenalidomide induces ubiquitination and degradation of CK1alpha in del(5q) MDS. Nature.

[31] Smyth G.K. (2004). Linear models and empirical bayes methods for assessing differential expression in microarray experiments. Stat. Appl. Genet. Mol. Biol..

[32] Yu Y., Yoon S.O., Poulogiannis G., Yang Q., Ma X.M., Villen J., Kubica N., Hoffman G.R., Cantley L.C., Gygi S.P. (2011). Phosphoproteomic analysis identifies Grb10 as an mTORC1 substrate that negatively regulates insulin signaling. Science.

[33] Bosbach B., Rossi F., Yozgat Y., Loo J., Zhang J.Q., Berrozpe G., Warpinski K., Ehlers I., Veach D., Kwok A. (2017). Direct engagement of the PI3K pathway by mutant KIT dominates oncogenic signaling in gastrointestinal stromal tumor. Proc. Natl. Acad. Sci. USA.

[34] Hsu P.P., Kang S.A., Rameseder J., Zhang Y., Ottina K.A., Lim D., Peterson T.R., Choi Y., Gray N.S., Yaffe M.B. (2011). The mTOR-regulated phosphoproteome reveals a mechanism of mTORC1-mediated inhibition of growth factor signaling. Science.

[35] Huttlin E.L., Jedrychowski M.P., Elias J.E., Goswami T., Rad R., Beausoleil S.A., Villen J., Haas W., Sowa M.E., Gygi S.P. (2010). A tissue-specific atlas of mouse protein phosphorylation and expression. Cell.

[36] Drake J.M., Graham N.A., Stoyanova T., Sedghi A., Goldstein A.S., Cai H., Smith D.A., Zhang H., Komisopoulou E., Huang J. (2012). Oncogene-specific activation of tyrosine kinase networks during prostate cancer progression. Proc. Natl. Acad. Sci. USA.

[37] Masoumi-Moghaddam S., Amini A., Morris D.L. (2014). The developing story of Sprouty and cancer. Cancer Metastasis Rev..

[38] Raynor J., Chi H. (2018). Sprouty branches out to control T cell memory. Proc. Natl. Acad. Sci. USA.

[39] Gross I., Bassit B., Benezra M., Licht J.D. (2001). Mammalian sprouty proteins inhibit cell growth and differentiation by preventing ras activation. J. Biol. Chem..

[40] Gaudet P., Livstone M.S., Lewis S.E., Thomas P.D. (2011). Phylogenetic-based propagation of functional annotations within the Gene Ontology consortium. Brief Bioinform..

[41] Blumer J., Rey J., Dehmelt L., Mazel T., Wu Y.W., Bastiaens P., Goody R.S., Itzen A. (2013). RabGEFs are a major determinant for specific Rab membrane targeting. J. Cell. Biol..

[42] Cherfils J., Zeghouf M. (2013). Regulation of small GTPases by GEFs, GAPs, and GDIs. Physiol. Rev..

[43] Wiza C., Nascimento E.B., Linssen M.M., Carlotti F., Herzfeld de Wiza D., van der Zon G.C., Maassen J.A., Diamant M., Guigas B., Ouwens D.M. (2013). Proline-rich Akt substrate of 40-kDa contains a nuclear export signal. Cell. Signal..

[44] Yang H., Jiang X., Li B., Yang H.J., Miller M., Yang A., Dhar A., Pavletich N.P. (2017). Mechanisms of mTORC1 activation by RHEB and inhibition by PRAS40. Nature.

[45] Fleissner F., Jazbutyte V., Fiedler J., Gupta S.K., Yin X., Xu Q., Galuppo P., Kneitz S., Mayr M., Ertl G. (2010). Short communication: asymmetric dimethylarginine impairs angiogenic progenitor cell function in patients with coronary artery disease through a microRNA-21-dependent mechanism. Circ. Res..

[46] Kim K., Ryu D., Dongiovanni P., Ozcan L., Nayak S., Ueberheide B., Valenti L., Auwerx J., Pajvani U.B. (2017). Degradation of PHLPP2 by KCTD17, via a Glucagon-Dependent Pathway, Promotes Hepatic Steatosis. Gastroenterology.

[47] Grzechnik A.T., Newton A.C. (2016). PHLPPing through history: a decade in the life of PHLPP phosphatases. Biochem. Soc. Trans..

[48] Slepak T.I., Salay L.D., Lemmon V.P., Bixby J.L. (2012). Dyrk kinases regulate phosphorylation of doublecortin, cytoskeletal organization, and neuronal morphology. Cytoskeleton (Hoboken).

[49] Nishi H., Hashimoto K., Panchenko A.R. (2011). Phosphorylation in protein-protein binding: effect on stability and function. Structure.

[50] Good M.C., Zalatan J.G., Lim W.A. (2011). Scaffold proteins: Hubs for controlling the flow of cellular information. Science.

[51] Giurgiu M., Reinhard J., Brauner B., Dunger-Kaltenbach I., Fobo G., Frishman G., Montrone C., Ruepp A. (2019). CORUM: The comprehensive resource of mammalian protein complexes-2019. Nucleic Acids Res..

[52] Ahn H.J., Tomura M., Yu W.G., Iwasaki M., Park W.R., Hamaoka T., Fujiwara H. (1998). Requirement for distinct Janus kinases and STAT proteins in T cell proliferation versus IFN-gamma production following IL-12 stimulation. J. Immunol..

[53] Papin J.A., Hunter T., Palsson B.O., Subramaniam S. (2005). Reconstruction of cellular signalling networks and analysis of their properties. Nat. Rev. Mol. Cell Biol..

[54] Straussman R., Morikawa T., Shee K., Barzily-Rokni M., Qian Z.R., Du J., Davis A., Mongare M.M., Gould J., Frederick D.T. (2012). Tumour micro-environment elicits innate resistance to RAF inhibitors through HGF secretion. Nature.

[55] Paulsson J., Ryden L., Strell C., Frings O., Tobin N.P., Fornander T., Bergh J., Landberg G., Stal O., Ostman A. (2017). High expression of stromal PDGFRbeta is associated with reduced benefit of tamoxifen in breast cancer. J. Pathol. Clin. Res..

[56] Kraman M., Bambrough P.J., Arnold J.N., Roberts E.W., Magiera L., Jones J.O., Gopinathan A., Tuveson D.A., Fearon D.T. (2010). Suppression of antitumor immunity by stromal cells expressing fibroblast activation protein-alpha. Science.

[57] Tan W., Zhang W., Strasner A., Grivennikov S., Cheng J.Q., Hoffman R.M., Karin M. (2011). Tumour-infiltrating regulatory T cells stimulate mammary cancer metastasis through RANKL-RANK signalling. Nature.

[58] Feig C., Jones J.O., Kraman M., Wells R.J., Deonarine A., Chan D.S., Connell C.M., Roberts E.W., Zhao Q., Caballero O.L. (2013). Targeting CXCL12 from FAP-expressing carcinoma-associated fibroblasts synergizes with anti-PD-L1 immunotherapy in pancreatic cancer. Proc. Natl. Acad. Sci. USA.

[59] Denton A.E., Roberts E.W., Linterman M.A., Fearon D.T. (2014). Fibroblastic reticular cells of the lymph node are required for retention of resting but not activated CD8+ T cells. Proc. Natl. Acad. Sci. USA.

[60] Yang X., Lin Y., Shi Y., Li B., Liu W., Yin W., Dang Y., Chu Y., Fan J., He R. (2016). FAP Promotes Immunosuppression by Cancer-Associated Fibroblasts in the Tumor Microenvironment via STAT3-CCL2 Signaling. Cancer Res..

[61] Toullec A., Gerald D., Despouy G., Bourachot B., Cardon M., Lefort S., Richardson M., Rigaill G., Parrini M.C., Lucchesi C. (2010). Oxidative stress promotes myofibroblast differentiation and tumour spreading. EMBO Mol. Med..

[62] Benyahia Z., Dussault N., Cayol M., Sigaud R., Berenguer-Daize C., Delfino C., Tounsi A., Garcia S., Martin P.M., Mabrouk K. (2017). Stromal fibroblasts present in breast carcinomas promote tumor growth and angiogenesis through adrenomedullin secretion. Oncotarget.

[63] Rasanen K., Vaheri A. (2010). Activation of fibroblasts in cancer stroma. Exp. Cell Res..

[64] Clayton A., Evans R.A., Pettit E., Hallett M., Williams J.D., Steadman R. (1998). Cellular activation through the ligation of intercellular adhesion molecule-1. J. Cell. Sci..

[65] Templeton A.J., Diez-Gonzalez L., Ace O., Vera-Badillo F., Seruga B., Jordan J., Amir E., Pandiella A., Ocana A. (2014). Prognostic relevance of receptor tyrosine kinase expression in breast cancer: a meta-analysis. Cancer Treat. Rev..

[66] Gruosso T., Gigoux M., Manem V.S.K., Bertos N., Zuo D., Perlitch I., Saleh S.M.I., Zhao H., Souleimanova M., Johnson R.M. (2019). Spatially distinct tumor immune microenvironments stratify triple-negative breast cancers. J. Clin. Investig..

[67] Bauer M., Su G., Casper C., He R., Rehrauer W., Friedl A. (2010). Heterogeneity of gene expression in stromal fibroblasts of human breast carcinomas and normal breast. Oncogene.

[68] Wagner J., Rapsomaniki M.A., Chevrier S., Anzeneder T., Langwieder C., Dykgers A., Rees M., Ramaswamy A., Muenst S., Soysal S.D. (2019). A Single-Cell Atlas of the Tumor and Immune Ecosystem of Human Breast Cancer. Cell.

[69] Costa A., Kieffer Y., Scholer-Dahirel A., Pelon F., Bourachot B., Cardon M., Sirven P., Magagna I., Fuhrmann L., Bernard C. (2018). Fibroblast Heterogeneity and Immunosuppressive Environment in Human Breast Cancer. Cancer Cell.

[70] Basson M.A., Akbulut S., Watson-Johnson J., Simon R., Carroll T.J., Shakya R., Gross I., Martin G.R., Lufkin T., McMahon A.P. (2005). Sprouty1 is a critical regulator of GDNF/RET-mediated kidney induction. Dev. Cell.

[71] Shim K., Minowada G., Coling D.E., Martin G.R. (2005). Sprouty2, a mouse deafness gene, regulates cell fate decisions in the auditory sensory epithelium by antagonizing FGF signaling. Dev. Cell.

[72] Klein O.D., Minowada G., Peterkova R., Kangas A., Yu B.D., Lesot H., Peterka M., Jernvall J., Martin G.R. (2006). Sprouty genes control diastema tooth development via bidirectional antagonism of epithelial-mesenchymal FGF signaling. Dev. Cell.

[73] Yang Y.H., Dudoit S., Luu P., Lin D.M., Peng V., Ngai J., Speed T.P. (2002). Normalization for cDNA microarray data: A robust composite method addressing single and multiple slide systematic variation. Nucleic Acids Res..

[74] Ting L., Cowley M.J., Hoon S.L., Guilhaus M., Raftery M.J., Cavicchioli R. (2009). Normalization and statistical analysis of quantitative proteomics data generated by metabolic labeling. Mol. Cell. Proteom..

[75] Yu G., Wang L.G., Han Y., He Q.Y. (2012). clusterProfiler: An R package for comparing biological themes among gene clusters. OMICS.

