# Supplementary material for: Quantitative Phosphoproteomics Reveals System-Wide Phosphorylation Network Altered by Spry in Mouse Mammary Stromal Fibroblasts
Source: Int J Mol Sci. 2019 Oct 30;20(21):5400. doi: 10.3390/ijms20215400 (PMC6862705; doi:10.3390/ijms20215400)
Supplement: Supplementary file 1 [file ijms-20-05400-s001.zip › Supplementary Figures.docx]

**Supplementary Figures S1-S3**


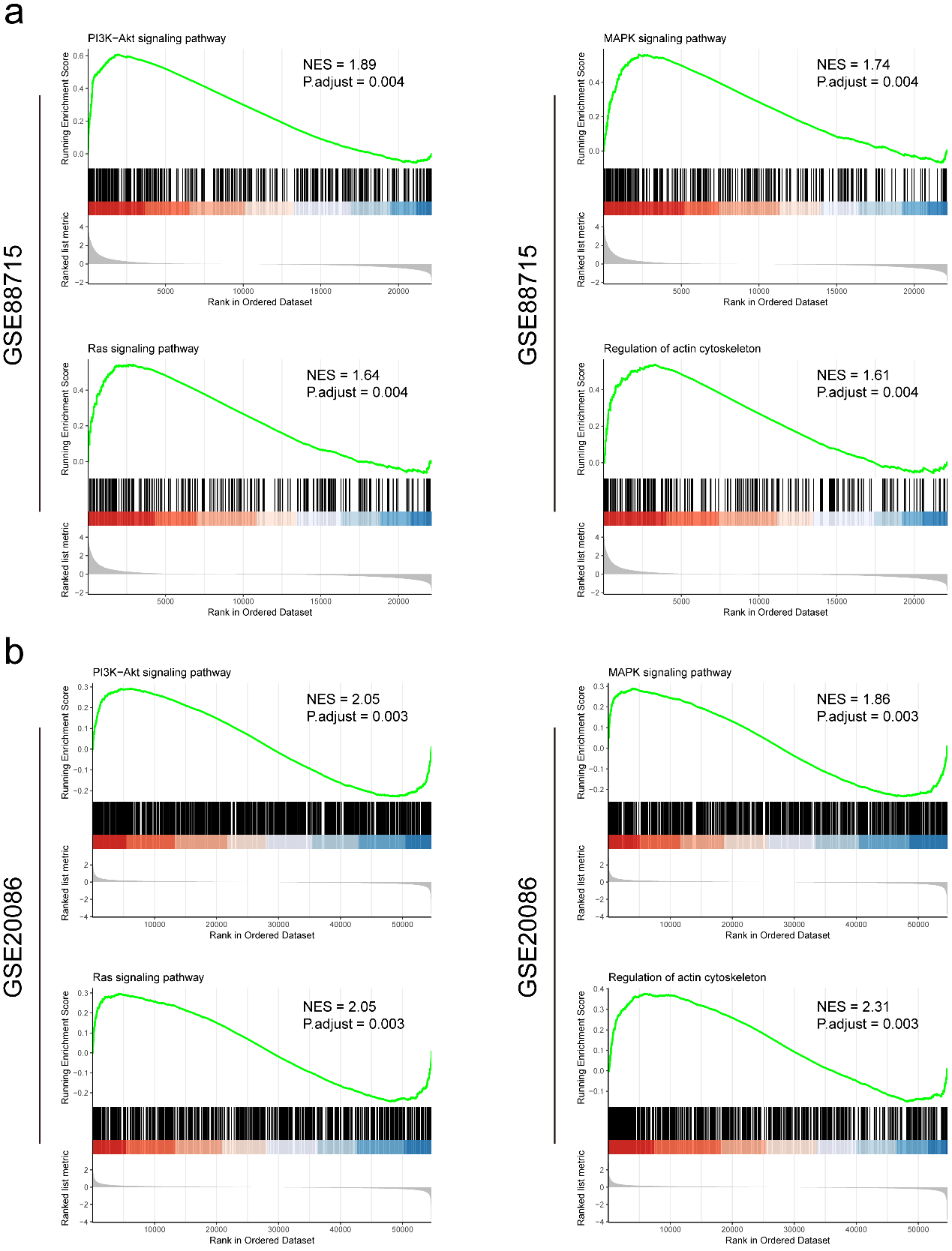


**Fig. S1. Several RTK downstream signaling pathways were enhanced in human breast cancer stromal fibroblasts.** PI3K-AKT signaling, MAPK signaling, RAS signaling and Regulation of actin cytoskeleton were enriched in human breast cancer stromal/fibroblasts in two open-access GEO datasets: GSE88715 (**a**) and GSE20086 (**b**).

**
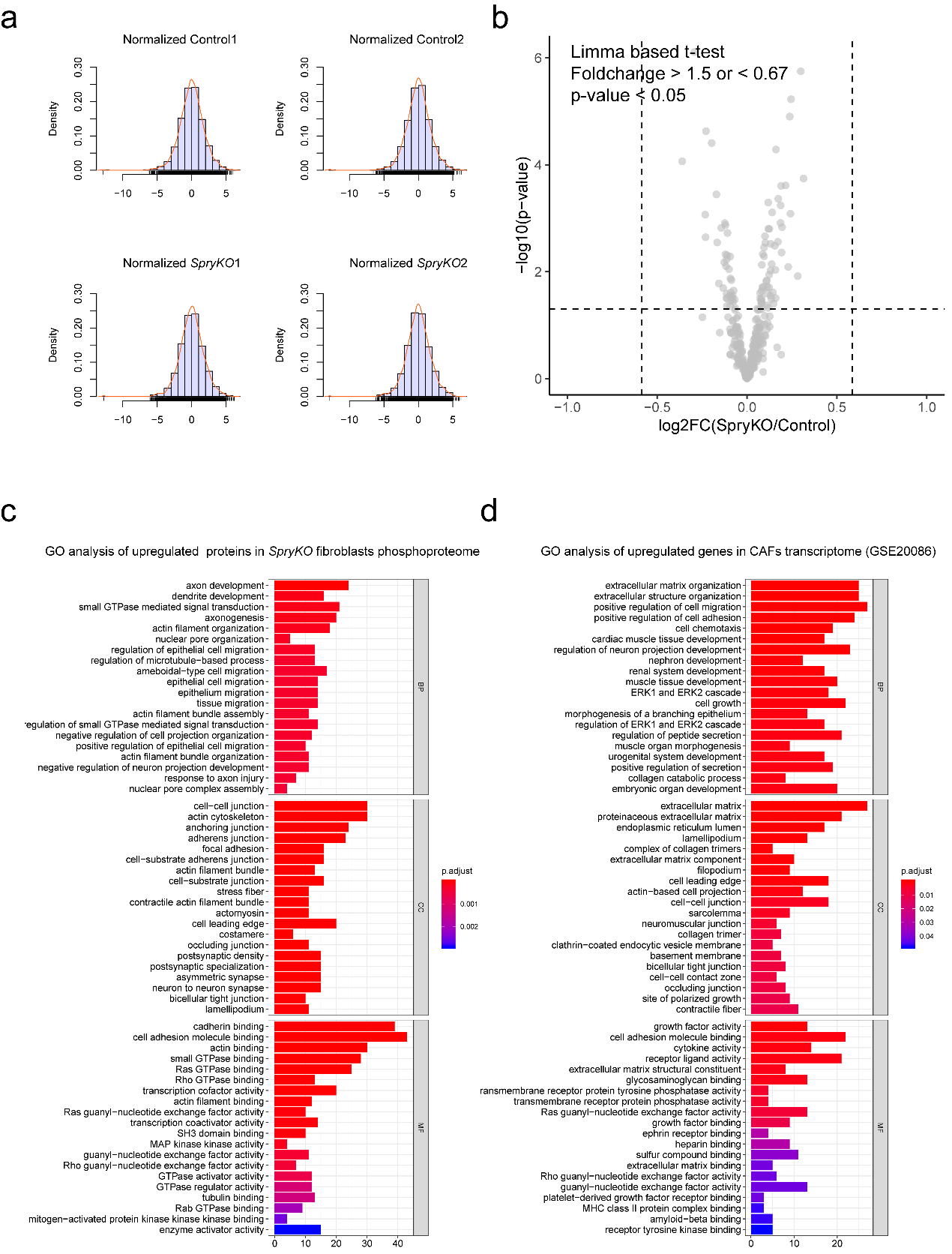
**

**Fig. S2. SPRY deficient mammary stromal fibroblasts show similar GO profiles to breast CAFs.**

1. Histograms of the data distribution of the two biological replicates in the phosphoproteome.
2. Quantitative proteomics analysis of the total protein expression level in control and *SpryKO* mouse mammary fibroblasts. 377 proteins of the 425 regulated phosphorylated proteins are shown (grey points).

**c**, **d**. GO enriched profiles of up-regulated phosphoproteins or genes in *SpryKO* fibroblasts (c) and breast CAFs (d). The color of the bars indicates the adjusted p-value of each term. Adjusted p-values of enriched terms are less than 0.05 and the top 20 terms are shown. (BP: biological process; CC: cellular components; MF: molecular function)


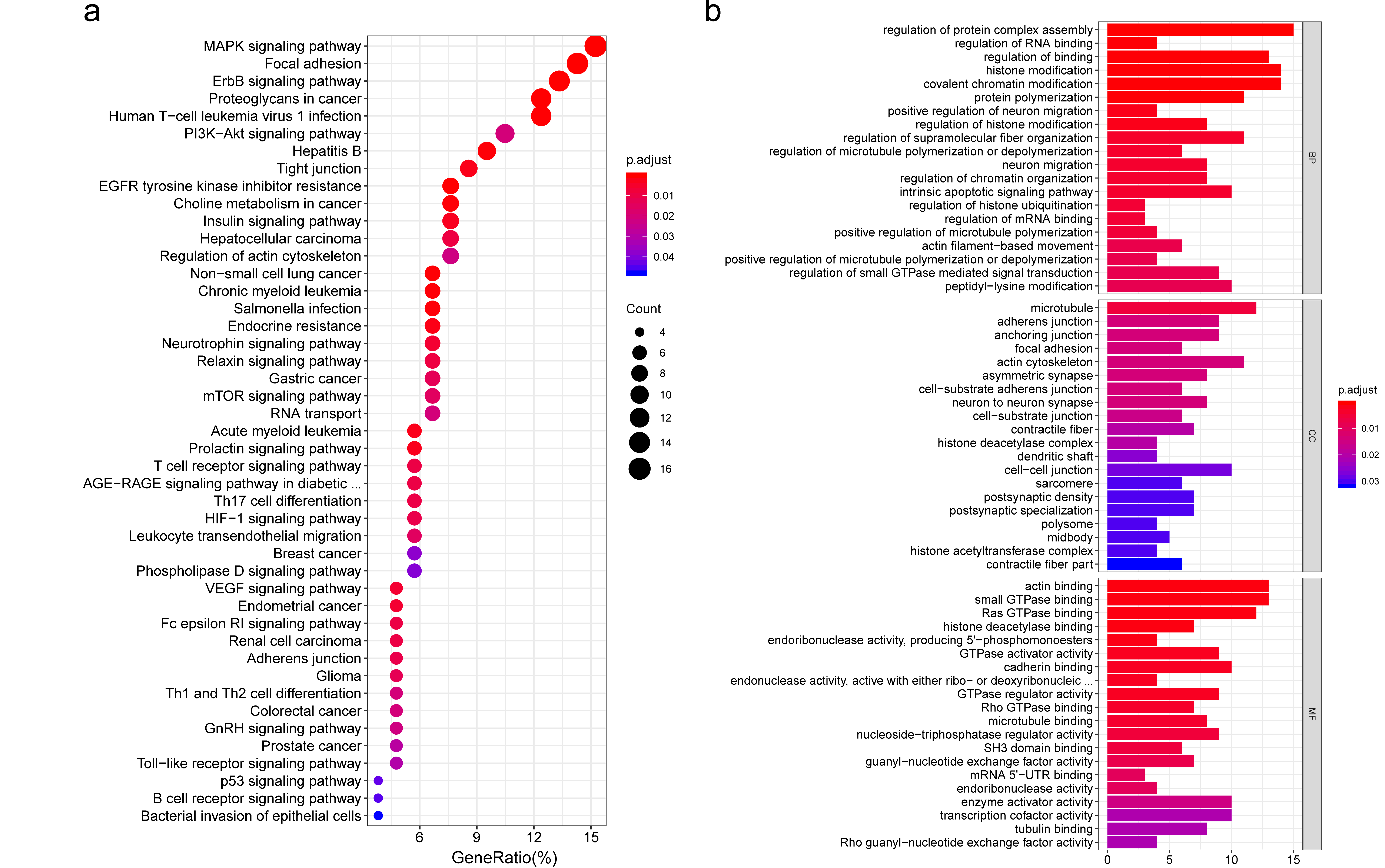


**Fig. S3. KEGG pathway analysis of up-regulated phosphoproteins and GO enrichment analysis of down-regulated phosphoproteins in *SpryKO* fibroblasts.**

1. KEGG pathway analysis of up-regulated phosphoproteins in *SpryKO* fibroblasts. Pathways are ranked by the number of enriched gene counts. The sizes of the dots indicate the number of gene counts and the color indicates the adjusted p-value of each term.
2. GO enrichment analysis of down-regulated phosphoproteins in *SpryKO* fibroblasts. The color of the bars indicates the adjusted p-value of each term. Adjusted p-values of enriched terms are less than 0.05 and the top 20 terms are shown. (BP: biological process; CC: cellular components; MF: molecular function)
