# Supplementary figures and images for: Quantitative Phosphoproteomics Reveals System-Wide Phosphorylation Network Altered by Spry in Mouse Mammary Stromal Fibroblasts
Source: Int J Mol Sci. 2019 Oct 30;20(21):5400. doi: 10.3390/ijms20215400 (PMC6862705; doi:10.3390/ijms20215400)

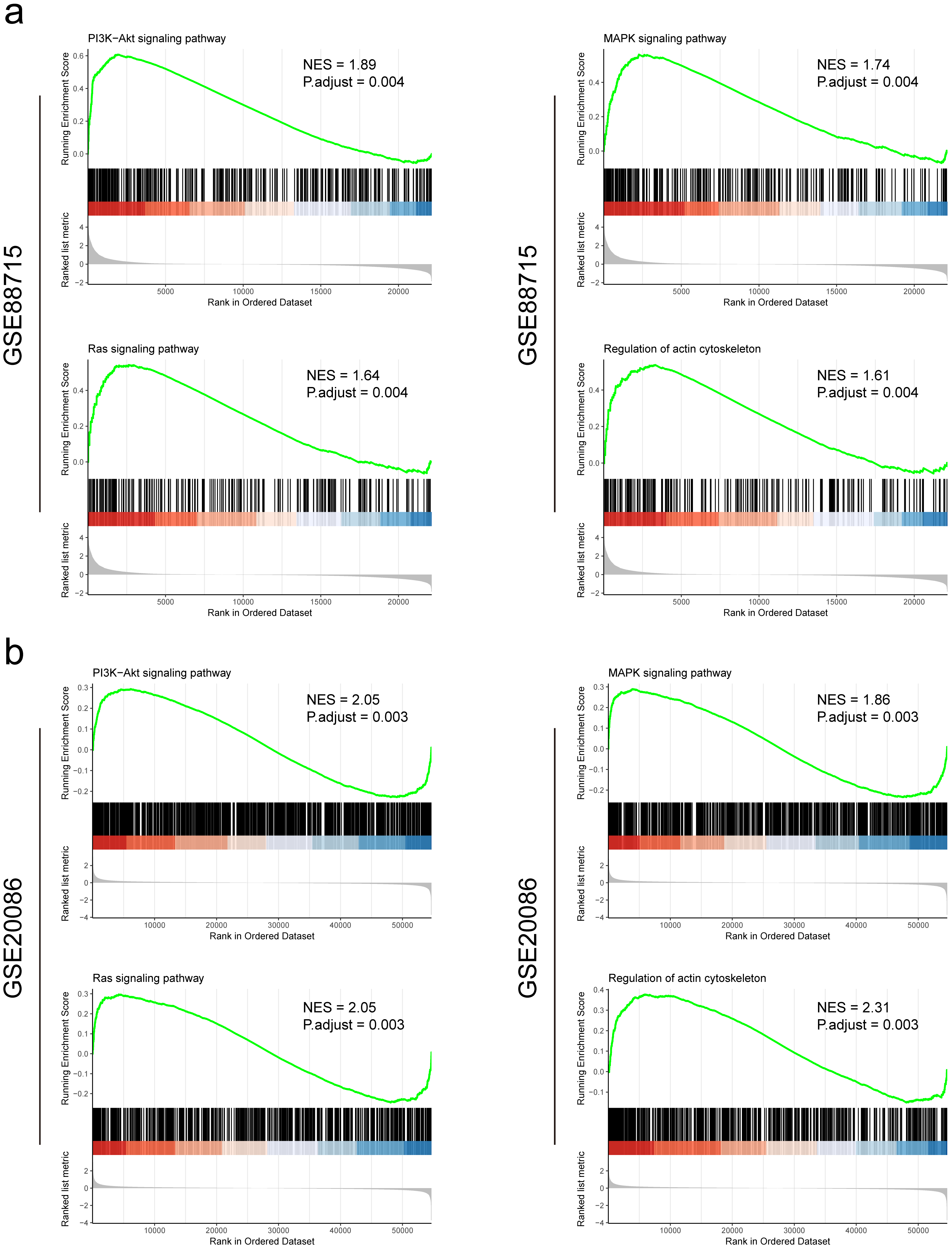

Supplement: Supplementary file 1 [file ijms-20-05400-s001.zip › FigS1.tif]

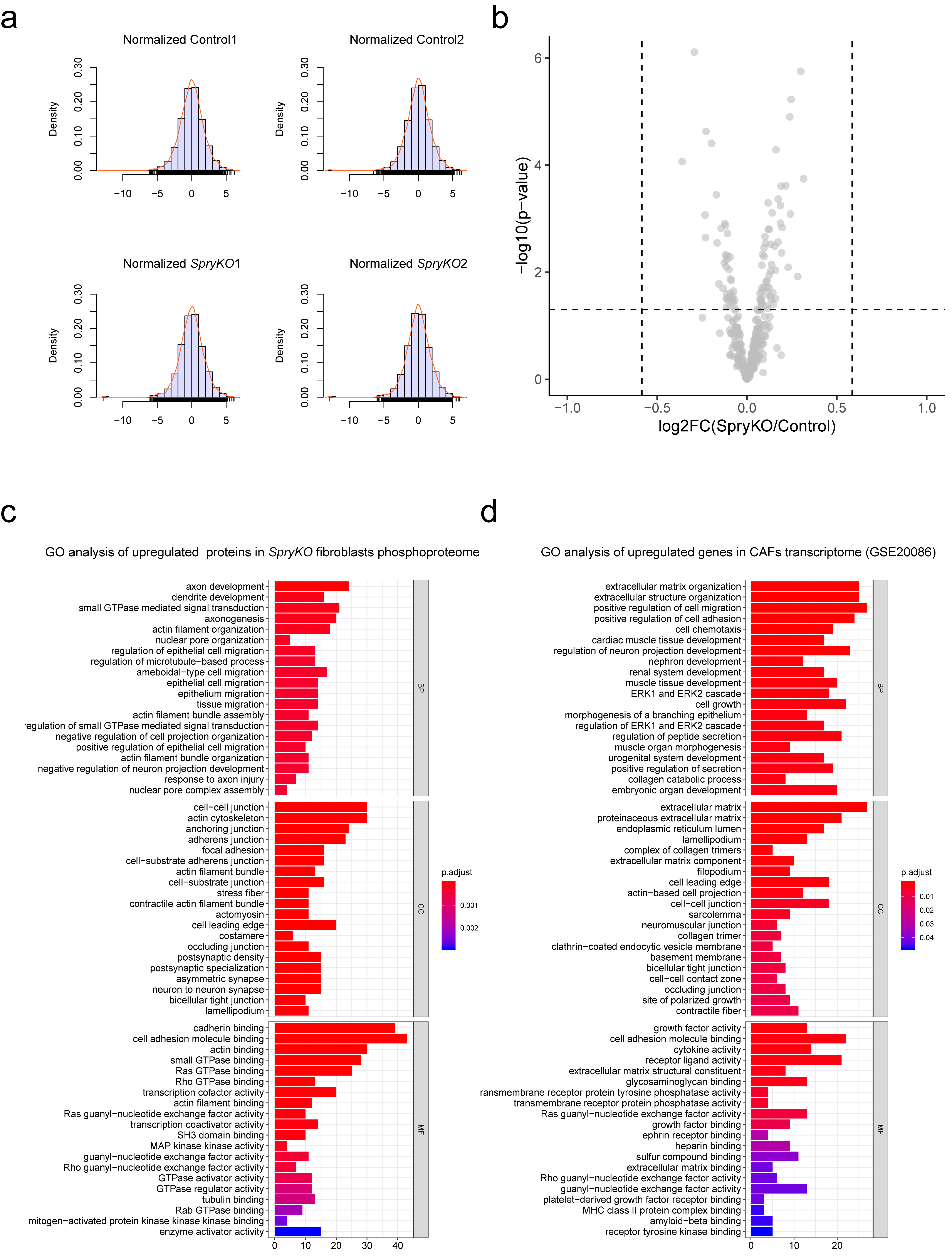

Supplement: Supplementary file 1 [file ijms-20-05400-s001.zip › FigS2.tif]

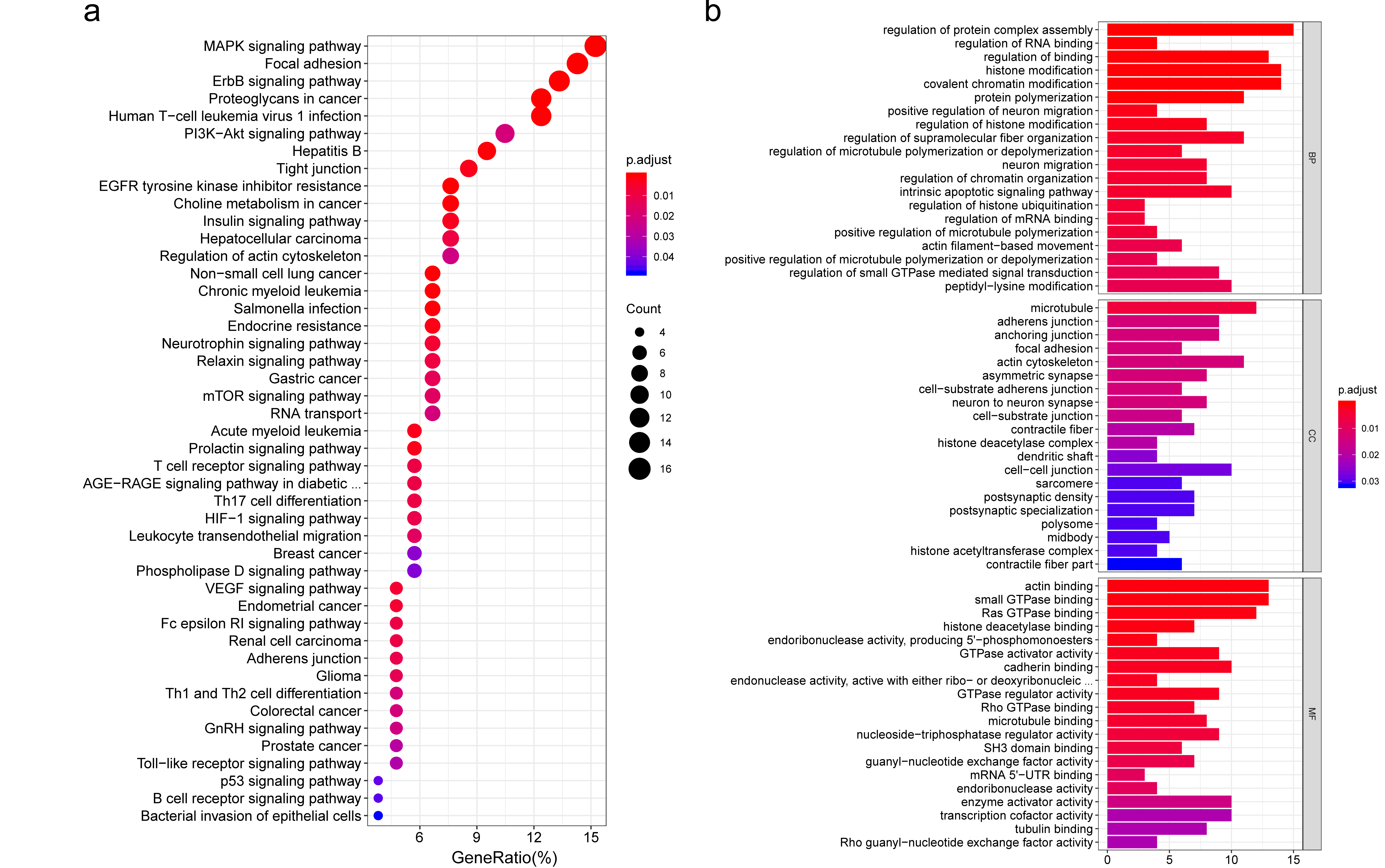

Supplement: Supplementary file 1 [file ijms-20-05400-s001.zip › FigS3.tif]
